# Habitat quality affects the incidence of morphological abnormalities in the endangered salamander *Ambystoma ordinarium*

**DOI:** 10.1371/journal.pone.0183573

**Published:** 2017-08-28

**Authors:** Carlos Soto-Rojas, Ireri Suazo-Ortuño, José Arturo Montoya Laos, Javier Alvarado-Díaz

**Affiliations:** 1 Instituto de Investigaciones sobre los Recursos Naturales, Universidad Michoacana de San Nicolás de Hidalgo, Col. Nueva Esperanza, Morelia, Michoacán, México; 2 Departamento de Matemáticas, Universidad de Sonora, Col. Centro, C.P., Hermosillo, Sonora, México; Fred Hutchinson Cancer Research Center, UNITED STATES

## Abstract

Identification of early warning signals previous to the occurrence of population decline or extinction is a major challenge for the conservation of animal species. Prevalence of morphological abnormalities in a population can be one of these signals. We registered morphological abnormalities in the salamander *Ambystoma ordinarium*. We also evaluated the relation between habitat quality and the prevalence of abnormalities in this species. We used scores from rapid bioassessment protocols (RBPs) to assess the habitat quality of streams inhabited by *A*. *ordinarium*. A preliminary survey indicated that of 29 streams where this species has been historically registered, 13 might have few or no *A*. *ordinarium*. The association between habitat quality and the incidence of morphological abnormalities was evaluated in these 16 streams. Of 502 sampled individuals, 224 (44.62%) had at least one body abnormality. Of the 224 individuals with body abnormalities, 84 (37.5%) presented more than one abnormality. Of a total of 5,522 evaluated morphological characters, 344 (6.74%) were abnormal. Partial loss of gills and missing digits were the most frequent abnormalities. Results of a binomial logistic regression indicated that the probability of a character of an individual to be abnormal was significantly associated with habitat quality; as the levels of the quality of the habitat increased, the prevalence of morphological abnormalities decreased. These results suggest that RBPs are a quick and useful method for assessing the habitat quality of streams inhabited by *A*. *ordinarium*. Given that RBPs provide rapid and cost-effective assessments of the ecological health of aquatic ecosystems, it will be important to test if the RBPs protocols can be used to rapidly assess habitat quality for other species of stream amphibians. The negative association between habitat quality and the prevalence of morpohological abnormalities that we found indicates that habitat condition plays an important role in the high number of abnormalities registered in *A*. *ordinarium*. Therefore, our results suggest that one of the several negative effects of habitat degradation on amphibians is an increase in the frequency of morphological abnormalities with marked consequences for the survival and general fitness of aquatic amphibians.

## Introduction

The loss of amphibian populations is a well-known example of the biodiversity crisis presently occurring on a global scale [1]. A number of factors, such as habitat loss and degradation, the introduction of invasive species, environmental contamination, diseases and parasites, unsustainable use and global climate change, have been recognized as causes of amphibian declines [2]. In association with this amphibian decline, an apparent increase in morphologic abnormalities has also been registered [3]. In most vertebrates, the presence of morphological abormalities among individuals of a population is frequently associated with mutations, developmental flaws and traumas. In addition, the background incidence of abnormailities in wild vertebrate populations ranges between 0 and 5% [4], although higher incidences of abnormalities have been reported in some species of amphibians (10–50%) [5, 6]. Johnson et al. [5] define abnormalities as any gross deviation from the normal range of morphological variation, including malformations (permanent structural defects resulting from abnormal development) and deformities (alterations to an otherwise correctly formed structure). In amphibians, these types of abnormalities frequently involve loss of digits or portions of limbs [1]. These abnormalities affect an individual’s fitness since their occurrence increases susceptibility to predation and lowers the individual’s efficiency in prey capture and mate acquisition [7]. Moreover, the energy assigned for repairing of abnormalities limits the availability of energy needed for other vital functions, such as feeding and reproduction [8]. An increase in the frequency of morphological abnormalities in amphibians has been associated with a number of environmental stressors, including ultraviolet radiation (UVB), pesticides, endoparasitosis, chemical contamination, damage caused by intraspecific interactions as well as by predation attempts [9, 10]. Even though various studies report a marked increment in the incidence of morphological abnormalities in amphibians during the last decades [1, 11], there are relatively few studies (e.g. [12, 13]) that evaluate the relationship between habitat disturbance and the incidence of abnormalities.

The Michoacan stream salamander (*Ambystoma ordinarium*) is a facultatively paedomorphic ambystomatid species inhabiting mountain streams in coniferous forests in the central Trans-Mexican Volcanic Belt. Localities where this species has been registered are situated between the vicinity of Patzcuaro Lake in the state of Michoacan and Tianguistenco in the western portion of the state of México [14]. The total occupation range of this salamander is less of 500 km^2^, and it is designated as an endangered species by the IUCN (www.iucnredlist.org accessed February 9, 2017). *A*. *ordinarium* is considered as a species in the category of Special Protection by the Mexican government (AmphibiaWeb; accessed February 12, 2017).

Considering that identifying environmental factors associated with the reported increment in morphological abnormalities is important for amphibian conservation [7, 15] and that the streams inhabited by *A*. *ordinarium* present different levels of habitat disturbance, our main aim in this study was to evaluate the role of habitat quality in the incidence of morphological abnormalities (sensu [5]) in this salamander. We addressed the following questions: 1) what types of morphological abnormalities are present in wild populations of *A*. *ordinarium*?, 2) is the method of rapid biossessment protocols (RBPs) of Barbour et al. [16] a reliable predictor of habitat quality for *A*. *ordinarium*?, and 3) what is the association between morphological abnormalities of *A*. *ordinarium* and habitat quality? We hypothesized that in streams with high disturbance levels, incidence of morphological abnormalities will be greater than in streams of low disturbance levels. Although in some species of amphibians morphological abnormalities have been linked to bite injuries inflicted among conspecifics during development (e.g. [6]), and therefore more abnormalities may be expected in productive high quality habitats, we hypothesized that incidence of morphological abnormalities will be greater in streams with high disturbance levels. This predicition was formulated by considering that the greater levels of habitat structural complexity in higher quality streams occupied by *A*. *ordinarium* provide numerous and diverse refuges [17], protecting individuals from bites potentially inflicted by conspecifics and aquatic predators.

## Materials and methods

### Ethics statement

This study was carried out in strict accordance with the guidelines for use of live amphibians and reptiles in field research compiled by the American Society of Ichthyologists and Herpetologists (ASIH). All procedures with animals were approved by the Committee on Biosafety and Bioethics (CBB) of the Institute of Chemical Biology of the Universidad Michoacana de San Nicolás de Hidalgo. In the research protocol, adult salamanders were visually detected and captured, anesthetized (by immersion in a solution of tricaine methanesulfonate, 0.25 g/l diluted in distilled water for an average of 15 minutes) and evaluated; they then recovered from anesthesia and were released the same day at the site of capture. The biological study, the protocol and the final report were overseen by the CBB.

### Field surveys

**Fig 1:**
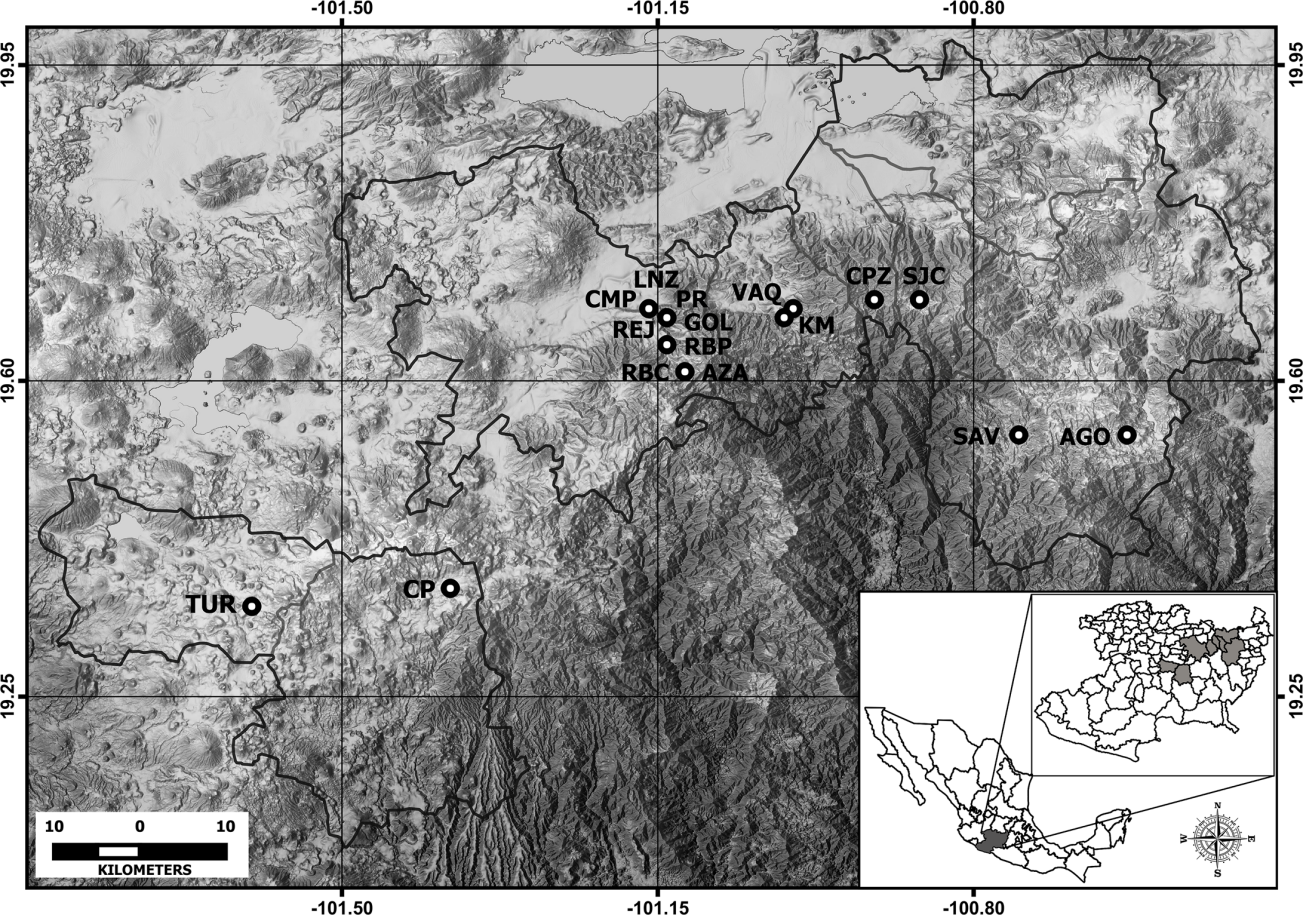
Distribution of streams sampled for morphological abnormalities of *A*. *ordinarium*. See Table 1 for acronyms.

**Fig 2:**
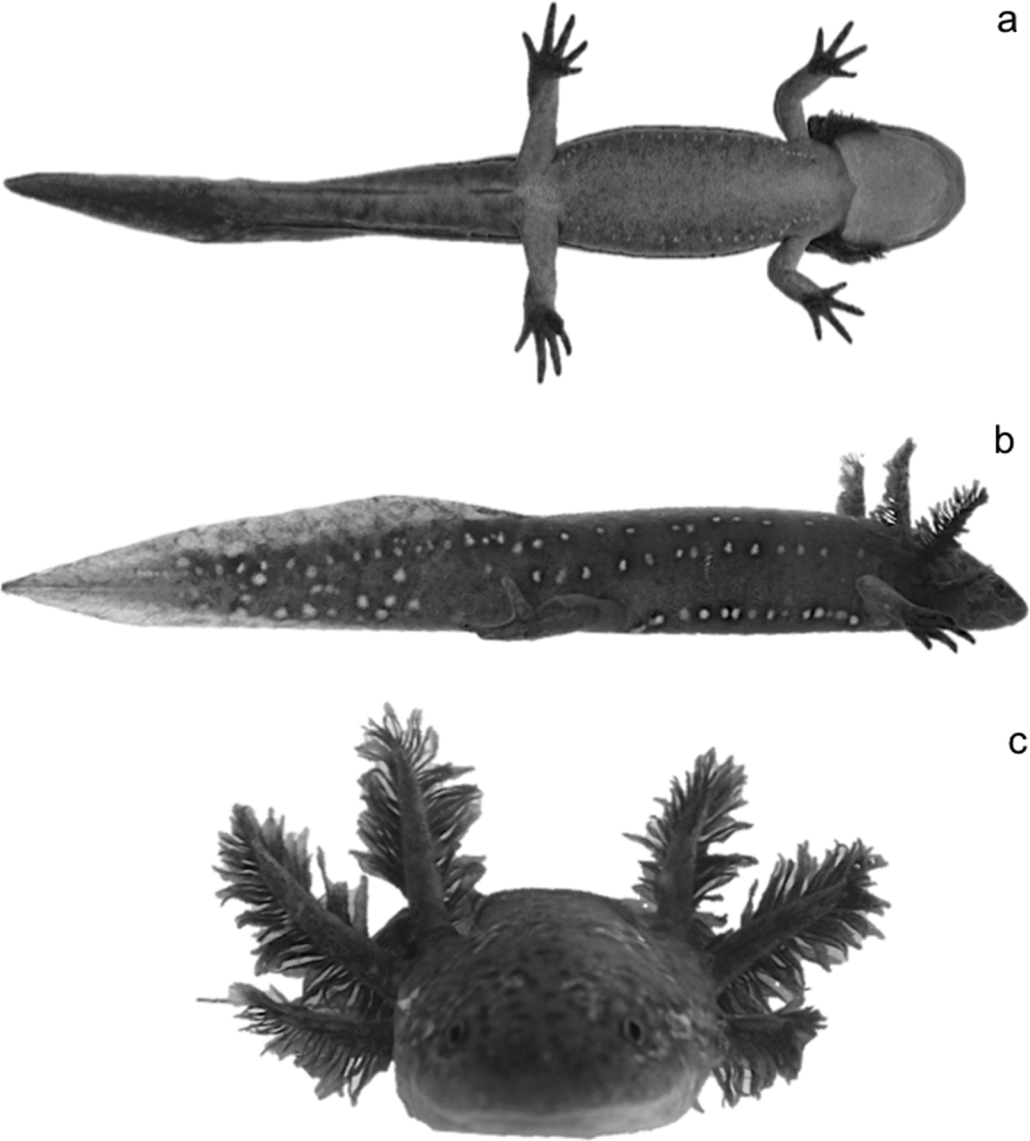
(a) ventral view, (b) lateral view, and (c) frontal view of the body of *A*. *ordinarium*.

We evaluated the presence of *A*. *ordinarium* in all 29 streams where it has been historically recorded [18, 19]. In each of these streams, a transect 800 m long was laid at the middle section, and salamanders were visually searched at the bottom of the stream by two persons for three hours. These surveys were carried out during February 2014. Sites correspond to mountain streams (altitude range = 1,950–3,400 m) in the central portion of the Trans-Mexican Volcanic Belt in Mexico [19, 20], immersed originally in pine, pine-oak, and fir forests. Anthropogenic disturbance inflicted on streams and adjacent areas includes bank erosion from trampling by vehicle traffic, cattle and people, selective logging of riparian vegetation, and conversion of upslope vegetation to suit agricultural activities [14]. In 16 of the 29 surveyed streams, we found populations of *A*. *ordinarium* (Fig 1). To collect field data on morphological abnormalities, surveys were performed during the dry season (March–July) of 2014. A 100 m long transect was laid at the middle section of each of the 16 streams where *A*. *ordinarium* populations existed. Each transect was sampled upstream three times (one time per day for three consecutive days). The sampling effort was standardized to 18 person/hours per transect (2 persons x 3 hours x 3 sampling days). Within each transect, salamanders were visually detected and captured with handheld nets capture (scientific collecting permit number SGPA/DGVS/04187/13). Once captured, salamanders were anesthetized by immersion in a solution of MS-222 (tricaine methanesulfonate, 0.25 g/l diluted in distilled water) for an average of 15 minutes [21, 22]. Only adult individuals (> 60 mm snout-vent length [18]) were considered in this study. Before the effect of anesthesia ended, salamanders were weighed (gr) (Primo Digital Scale), marked with a PIT (passive integrated transponder) tag (11.5 mm long and 2.1 mm in diameter by Trovan ID100A) following the technique of Winandy and Denoël [23] and photographed. PIT tagging was part of a complementary study to estimate population size of this species. In the present study, tags were used to avoid counting individuals more than once in the consecutive surveys. Photographs were used to identify morphological abnormalities. Photographs were taken of the venter, sides and front of the body (Fig 2). The same person took all photographs. Each image was referenced with an ID number. Once the procedure was completed and salamanders recovered from anesthesia, they were released the same day at the site of capture.

### Identifying morphological abnormalities

**Fig 3:**
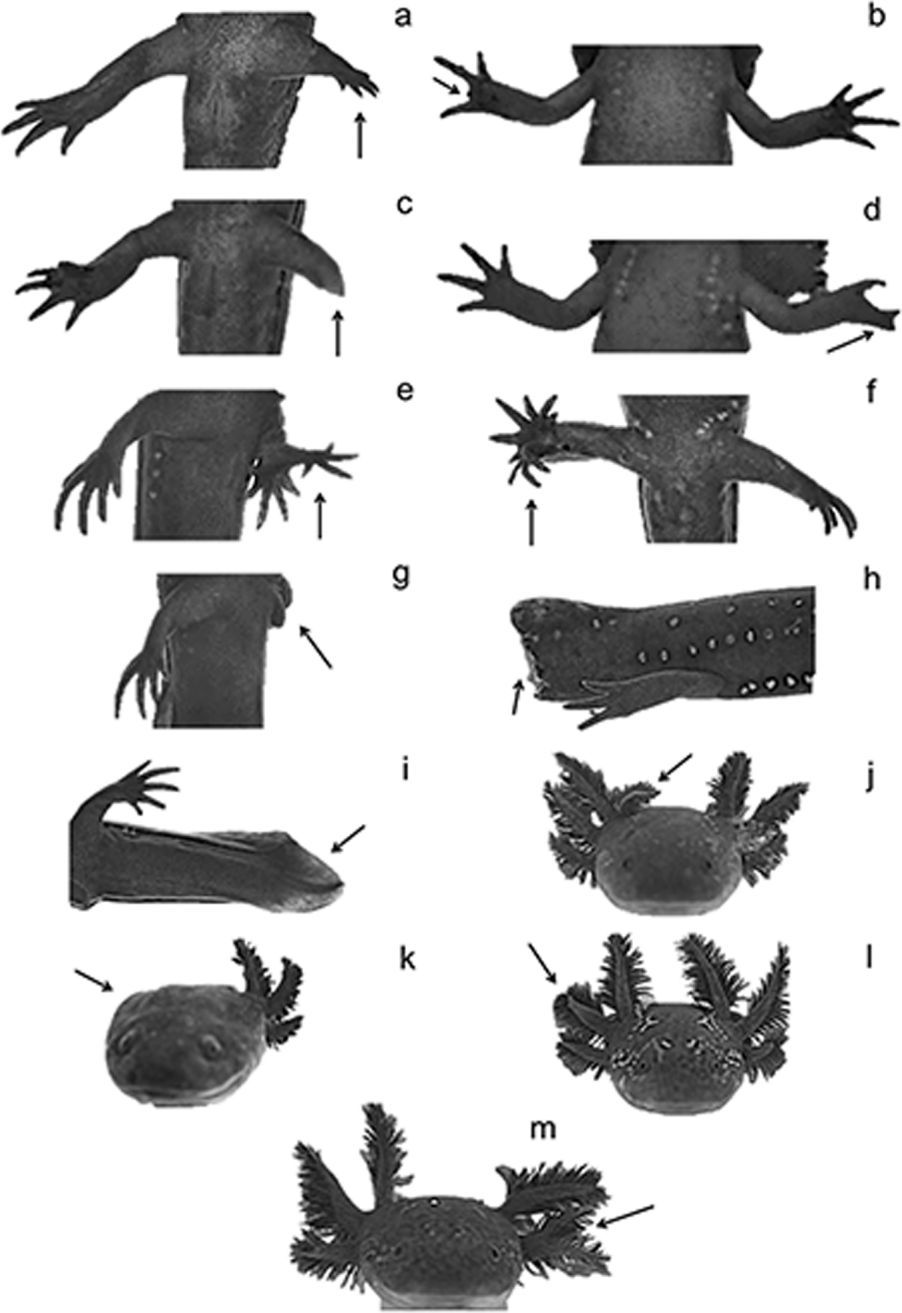
Types of morphological abnormalities identified in *A*. *ordinarium*: (a) micromelia, (b) ectrodactyly, (c) brachydactyly, (d) syndactyly, (e) polimely, (f) polydactyly, (g) ectromelia, (h) incomplete tail, (i) regenerated tail, (j) polygills, (k) ectrogills,(l) braquigills, (m) syngills.

Images were used to visually search for morphological abnormalities in the laboratory by the same person. Each abnormality of forelimbs, hindlimbs and tail was defined as follows: micromelia (short limbs), ectrodactyly (missing digits), brachydactyly (missing part of a limb), syndactyly (fusion of fingers), polymelia (extra number of limbs and extra number of hands or feet), polydactyly (extra number of digits), ectromelia (missing a limb), incomplete tail (missing part of tail), and regenerated tail [6, 15, 24, 25] (Fig 3). We used the following terms in referring to gill abnormalities: polygills (extra number of gills), ectrogills (missing gills), braquigills (missing part of a gill) and syngills (fusion of gills) (Fig 3). Images of the ventral body part were used to determine snout-vent length (SVL), length of tail and total body length by using the software ImageJ [22].

### Habitat quality

We used the rapid bioassessment protocols (RBPs) of Barbour et al. [16] to assess the habitat quality of all of the 29 surveyed streams where historically *A*. *ordinarium* has been registered. This protocol is a visually based method that evaluates habitat condition by using a categorical scoring system to appraise habitat condition [16]. Following the RBPs, the variables measured to assess habitat quality were the vegetative protection of streambank, type and embeddedness of substrate, channel flow status, patterns of velocity and depth, sediment deposition, riffle frequency, and human alterations. These variables were recorded along a 100 m long transect laid along the middle section of each stream. Scored values range from 0 (indicating a markedly disturbed condition) to 200 (indicating a habitat in pristine condition) [16]. The evaluated streams were assigned to four habitat condition categories according to RBPs scores: poor (0–59), marginal (60–112), suboptimal (113–165) and optimal (166–200).

### Physichochemical variables

To test if RBPs are a good predictor of habitat quality for *A*. *ordinarium*, we evaluated if the range of values of physicochemical and structural variables that are relevant for this species in its habitat [17] and that are not considered by the RBPs matched the categories of habitat condition considered by RBPs. This set of variables was found to be significant for the selection of aquatic microhabitats by *A*. *ordinarium* [17]. A 100 m long transect was laid in the middle of each of the 29 streams where the presence of *A*. *ordinarium* was historically reported. Every 10 m along the 100 m transect at sampled streams a gauging point was established. At each of these points, the following measurements were made: water temperature, conductivity and dissolved oxygen (by using a YSI 85,Yellow Spring, Ohio, USA), pH (with a conductronic PC18), water velocity and flow (with a flowmeter model FP211, Global Water), air temperature and humidity (measured at 1 m above water surface with a microstation Kestrel 4500), width and depth of the stream (with a tape measure) and canopy cover (with a densiometer).

### Analysis

**Fig 4:**
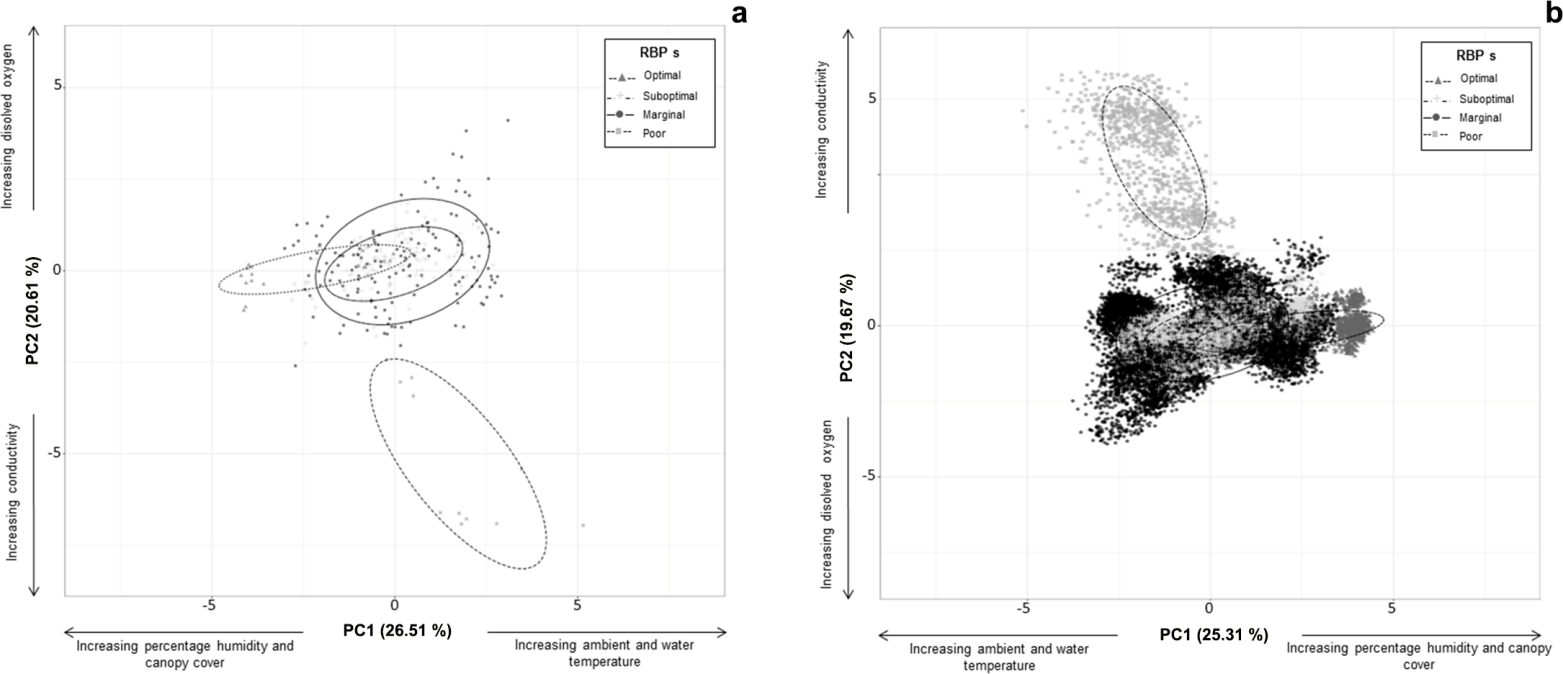
(a) Analysis of principal component (PCA) of physichochemical and structural variables of the streams where *A*. *ordinarium* has been historically recorded. Categories of habitat condition according to RBPs scores: optimal, suboptimal, marginal and poor and (b) bootstrap simulation of PCA results.

To assess if the the range of values of the physichochemical and structural variables that were measured, in addition to those considered by RBPs, corresponded to the categories of habitat condition established by RBPs (optimal, suboptimal, marginal, poor), we used a principal component analysis with R Core Team [26]. Previous to PCA analysis, we performed autoscaling of sampled variables. To estimate the distribution of categories of the habitat condition established by RBPs, we employed a non-parametric bootstrap approach. We resampled times the physichochemical and structural variables one thousand times at each sampled stream. The PCA showed that values of evaluated physichochemical and structural variables were separated, with sampling points in the inferior right quadrant corresponding to the most disturbed streams, and sampling points in the upper left quadrant corresponding to the most conserved streams (Fig 4A and 4B). A less defined cluster of points in the middle part of the figure corresponds to streams with intermediate conditions of disturbance (Fig 4A and 4B). The PCA ordination corresponded to labels of RBPs categories of habitat condition (Fig 4A and 4B). Therefore, to explore the relationship between morphological abnormalities and habitat quality we employed the scores of RBPs.

To assess the relationship between habitat quality and the probability of abnormalities for each body character, we considered eleven visible characters by individual: six gills (three on each side), four limbs (two forelimbs, two hindlimbs) and a tail. Therefore, a total of 5,522 body characters were evaluated (11 characters x 502 individuals). The evaluation of each character was independent, and each was registered as either abnormal (success) or normal (failure) (binary variable). Assuming that habitat quality is a factor that can directly or indirectly influence the health status of an organism, we used a binomial logistic regression model [27] to assess the relationship between habitat quality and the probability of abnormality of a body character. In this regression model, we used the values of RBPs as a variation factor. We assumed that the probability of a body character of an individual of the population to be abnormal was determined by habitat quality. This model is given in terms of the following:

$$Y_{i}∼Bin\;(N,P_{i,})$$

where

Yi = the number of body abnormalities (successes) in N = 11 body characters evaluated to the i-th individual, i = 1,…,502.

P_i_ = the probability that a character of an individual of the population is abnormal is modeled based on

$$P_{i}=\frac{e^{\alpha +\beta X_{i}}}{1+e^{\alpha +\beta X_{i}}},$$

where *X*_*i*_ is the RBPs score corresponding to habitat of the i-th individual and (*α*,*β*) is the vector of regression coefficients. The value of *α* yields probability when the RBPs score is zero. The coefficient *β* is the amount the logit (log-odds) changes with a one-unit change in RBPs score:

$$ln\left(\frac{P_{i}}{1-P_{i}}\right)=\alpha +\beta X_{i}.$$

## Results

### Habitat quality

The scores of RBPs indicated that two of the 29 sampled streams were in the optimal category of habitat condition (172–178), 10 in suboptimal condition (123–157), 14 in marginal condition (63–110) and three in poor condition (0.0–59) (Table 1, S1 Fig). PCA analysis showed that the physichochemical and structural variables (those not considered by RBPs) presented a continuous distribution (Fig 4A), with no single site segregating from the rest based on physichochemical and structural features of the habitat. The first two PCA components explained 47.7% of the variability in the data. In the first PCA component, streams with an optimal habitat condition presented a higher percentage of tree cover (-0.24) and higher values of air humidity (-0.50) compared to streams in the suboptimal, marginal and poor habitat categories. These streams in the lower categories of habitat condition presented higher water (0.44) and air temperatures (0.55). In general, in the second PCA component streams with a lower habitat condition presented higher conductivity (-0.47) and a lower concentration of dissolved oxygen (0.60) in the water. Bootstrap analysis showed an affinity of the distribution of physichochemical and structural data in relation to RBPs’ scores of habitat quality (Fig 4B).

**Table 1:** Scores of RBPs, habitat condition and the abundance of *A*. *ordinarium* in the 29 sampled streams where this species has been historically recorded.

				Coordinates		
Locality	Acronyms	RBPs scores	Habitat condition	Longitude	Latitude	Abundance
Agostitlán	AGO	156	Suboptimal	100°37'40.3''	19°32'9.8''	45
San josé de la Cumbre	SJC	172	Optimal	100°51'31.4''	19°41'11.''	78
San Antonio Villalongín	SAV	134	Suboptimal	100°44'54.9''	19°32'5.''	5
Carindapaz	CPZ	154	Suboptimal	100°54'25.7''	19°41'15.8.''	21
Kilómetro 23	KM	81	Marginal	101°0'5.1''	19°39'34.''	9
Campestre	CMP	63	Marginal	101°9'27.4''	19°40'51.4.''	50
Linezo	LNZ	75	Marginal	101°9'22.8''	19°40'45.9''	10
Piedra redonda	PR	125	Suboptimal	101°8'41''	19°40'3''	13
Cruz del Plato	CP	154	Suboptimal	101°16'58.7''	19°24'53.5''	22
Río Bello C	RBC	178	Optimal	101°7'25.7''	19°36'39.6''	32
Río Bello P	RBP	105	Marginal	101°8'17.2''	19°38'28.3''	80
Turirán	TUR	157	Suboptimal	101°35'54.1''	19°21'15.4''	26
Agua Zarca	AZA	153	Suboptimal	101°7'29.1''	19°36'49.8''	64
Golondrinas	GOL	123	Suboptimal	101°8'35''	19°39'58''	22
Vaquerito	VAQ	145	Suboptimal	101°0'7.6''	19°40'54.5''	12
Reja	REJ	110	Marginal	101°8'42.3''	19°40'14.04''	13
Puerto Garnica	PGA	85	Marginal	101°49'19.4''	19°40'38''	0
Pino Gordo	PGO	134	Suboptimal	100°47'56.7''	19°39'33.9''	0
Mil Cumbres	MCU	60	Marginal	100°46'27.8''	19°37'15.4''	0
Piedra redonda	PRE	16	Poor	101°0'48.6''	19°38'56.3''	0
Zurumbeneo	ZUR	87	Marginal	101°1'3.3''	19°41'57.7''	0
San Gregorio	SGR	66	Marginal	101°32'20.6''	19°24'3.6''	0
Paso del Muerto	PMU	101	Marginal	101°36'34.8''	19°19'29.2''	0
Tepetate	TEP	17	Poor	101°34'54.8''	19°22'22.9''	0
Cieneguillas	CIE	91	Marginal	100°44'58.5''	19°42'43''	0
Ojo de Agua	OAG	111	Marginal	100°45'13.4''	19°44'39.3''	0
Querendaro	QUE	83	Marginal	100°53'8''	19°48'28.7.3''	0
Los Sauces	SAU	91	Marginal	100°42'5.9''	19°40'3.8''	0
Iratzio	IRT	26	Poor	101°24'37.23''	19°38'31.74''	0

### Morphological abnormalities

Of the 502 individuals collected, 224 (44.62%) presented at least one morphological abnormality. Of these, 140 presented one abnormality, 60 two abnormalities, 22 three and 2 individuals presented four abnormalities. Of the total of 5,522 analyzed characters, 334 were abnormal (6.04%). Of the 334 abnormal characters, braquigills and ectrodactyly with an ocurrence of 26% and 23%, respectively, were the most frequent abnormalities, followed in descending order by micromelia (8.98%), incomplete tail (8.38%), poligills (8.08%), syndactyly (6.89%), ectrogills (5.39%), brachydactyly (4.19%), ectromelia (3.89%), polydactyly (1.80%), regenerated tail (1.50%), polymelia (0.90%), microdactyly (0.60) and syngills (0.30%).

### Probability of abnormalities

**Fig 5:**
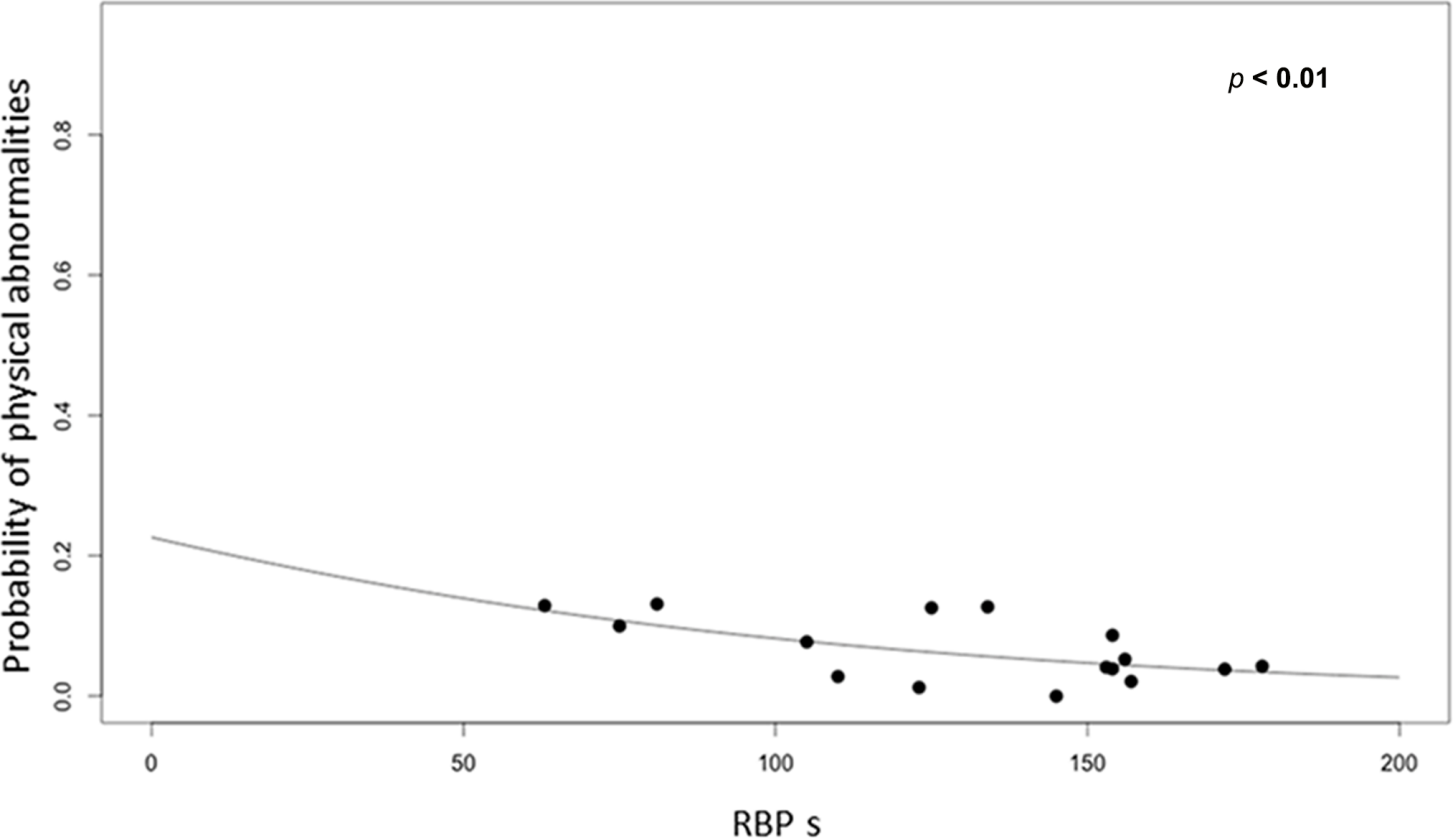
Results of the model of binomial logistic regression of the probability of the character of an individual of *A*. *ordinarium* being abnormal in relation to habitat quality (RBPs scores). The estimated parameters are α = -1.22, SD = 0.18, P-value < 0.01; β = - 0.01, SD = 0.001, P-value < 0.01 (S1 Table).

The results of the binomial logistic regression showed that the probability of a character of an individual of the population being abnormal was significantly associated with the scores of habitat condition of RBPs (P-value < 0.001, *β* = −0.01; *SD* = 0.001)). The higher the RBPs scores the lower the probability of individuals presenting morphological abnormalities (Fig 5).

## Discussion

The results of our survey of the 29 streams where historically *A*. *ordinarium* has been recorded must be taken with a certain level of caution. Failing to detect *A*. *ordinarium* in 13 of the 29 surveyed streams does not necessarily imply that the species has become extinct in these streams. This species is more sensitive to thyroid hormone-induced metamorphosis than other ambystomatids [6]. Considering that surveys were carried out to register larvae/and or paedomorphic individuals, it is possible that the detection of individuals was negatively affected if there was a higher proportion of metamorphic forms in streams where this species was not detected. An additional consideration in interpreting both the presence/absence of *A*. *ordinarium* and the incidence of morphological abnormalities is the short window of time (February for presence/absence and March-July for abnormalities) in which field surveys were performed. This period of time corresponds to the dry season in the study area and is when the mountain streams present lower depth and flow rate, as well as clearer water—conditions that allow a better detectability of salamanders in the streams. Therefore, possible seasonal variations in the incidence of abnormalities were not detected with our sampling design.

Understanding how animals in nature respond to the various anthropogenic environmental stressors that threaten their survival is critical for the planning of conservation strategies and actions [28]. Animal species, especially inhabitants of streams, such as the salamander *Ambystoma oridnarium*, are frequently threatened by human activities like urbanization, deforestation and agriculture that negatively affect habitat conditions [13, 29, 30]. Considering the fast pace at which habitats are being transformed, it is important to identify methods that provide rapid and confident assessments of the effects of modifications of habitat quality on the health of populations. In this study we found that the range of values of physicochemical and structural variables that are relevant for *A*. *ordinarium* in its natural habitat [17] is positively associated with the habitat condition categories of RBPs. Streams that according to scores of RBPs were of optimal habitat condition (high habitat quality) presented a higher percentage of canopy cover, lower values of water conductivity, lower water temperature and higher levels of dissolved oxygen than streams with lower scores of habitat condition. These results suggest that the RBPs of Barbour et al. [16] are a quick and useful method for assessing the habitat quality of streams inhabited by *A*. *ordinarium*. Given that RBPs provide rapid and cost-effective assessments of water quality and ecological health of aquatic ecosystems [31], it will be important to test if the RBPs protocols can be used to rapidly assess habitat quality for other species of stream amphibians.

Habitat quality is frequently studied in relation to abundance, density, richnness and diversity of species. Some of these measures, however, such as abundance and density may not be adequate predictors of the well-being and physiological performance of individuals [27, 32, 33]. Considering that morphological abnormalities in amphibians have been associated with various types of environmental stressors (e.g., chemical contamination, UBV radiation, infection of parasites, predation attempts), it is reasonable to expect a higher rate of abnormalities in habitats with low levels of environmental quality. Thererfore, our main hypothesis in this study was that we would find an association between habitat quality and prevalence of morphological abnormalities in *A*. *ordinarium*. Results supported this hypothesis since binomial regression analysis showed a negative association between habitat quality (as indicated by RBPs scores) and the probablility of morphological abnormalities. As the scores of habitat condition were higher, the probability of abnormalities became lower.

The negative association between habitat quality and the prevalence of morphological abnormalities that we found suggests that habitat condition plays an important role in the high number of abnormalities registered in *A*. *ordinarium*. Only two of the 29 surveyed streams were assigned to the optimal category of habitat condition according to RBPs scores. Ten streams were assigned to the suboptimal, 14 streams to the marginal and three streams to the poor categories of habitat condition. Therefore, 93% of the surveyed streams presented lower than optimal habitat conditions, resulting apparently in a high number of salamanders with morphological abnormalities.

Morphological abnormalities in amphibians have been frequently associated with a reduction in survival and fitness [34, 35]. The 44.6% of individuals of *A*. *ordinarium* with morphological abnormalities recorded in this study is markedly higher than the background incidence of 0 to 5.0% reported in some wild vertebrate populations in natural habitats [4] and near the higher end of the range of abnormalities reported in some amphibian species (10–50%) [6, 7]. Additionally, of the 224 individuals evaluated for abnormalities, 84 (37.5%) presented more than one abnormality. It is pertinent to acknowledge that in contrast to other studies we included gill abnormalities in the calculation of the 44.6% of sampled individuals with at least one type of abnormality. Even though the gill abnormalities recorded might have been in part related to different proportions of metamorphic forms in dissimilar habitats, if we substract from the estimation those individuals with gill abnormalities, the resultant figure (29.4%) is still markedly higher than the background reported incidence (0–5%).

Loss of digits has been reported as the most frequent morphological abnormality in amphibians (e.g. [5, 36]). To our knowledge, ours is the first study about wild amphibians that reports morphological abnormalities in gills. Braquigills, the partial loss of gills was the most frequent abnormality (26%), whereas ectrodactyly, the loss of digits presented an incidence of 23.0%. Gills in urodeles are responsible for up to 60–70% of oxygen uptake and CO2 removal [37]. Therefore, a reduction of gill surface as a result of injures likely affects the fitness of those individuals with this type of morphological abnormality.

Reported causes of morphological abnormalities in amphibians include road proximity [38], trematode parasites [39, 40], predation attacks [41], radiation [42] and chemical contaminants [43]. Our experimental design did not allow us to identify the relationship between the type of abnormalities and specific causative agents. The negative association between habitat quality and prevalence of abnormalities offered us the possibility of exploring the role of some possible causal factors.

The proximity of roads has been associated with the incidence of morphological abnormailities in frogs [38]. The most obvious effects are direct injury or death from contact with vehicles [44]. Of the streams that we surveyed, those with lower scores of habitat quality had paved or gravel roads parallel to or crossing the streams. Traffic on these roads included trucks, motorcycles, cars and bicycles. Cattle also trampled portions of the banks of these lower habitat quality streams. Both traffic crossing the bed of streams and trampling of banks by cattle are potential causes of some of the morphological abnormalities related to body injures, such as partial or total loss of gills, digits, extremities and tail. Roads also crossed some of the streams with better habitat quality scores, but elevated bridges were used for traffic passage.

Predation attempts have also been considered as one cause of body injures in aquatic amphibians [8]. Therefore, another possible source of abnormalities related to body injures is attacks by predators such as Odonata nymphs [14] and garter snakes (*Thamnophis cyrtopsis* [45]). Predatory fish are also one of the major predators of salamanders in aquatic escosystems [46] and can inflict injures such as partial or total loss of tail, digits, gills and extremities [47]. In three of the 16 streams sampled for prevalence of abnormalities, introduced rainbow trout (*Oncorhynchu mykiss*), a known predator of aquatic salamanders [46] was registered in this study.

Parasite infection is another possible cause of the morphological abnormalities recorded in this study. It has been reported that morphological abnormalities such as polymelia (extra number of limbs, hands or feet), polydactyly (extra number of digits), micromelia (short limbs) and syndactyly (fusion of digits) are associated with the presence of parasites such as trematodes (i.e. *Robeiroia sp*.) [7, 24, 36]. It has been suggested that the process of regeneration of limbs and digits in salamanders is sensitive to the effects of parasites, resulting in abnormal structures [48]. In our study, although of low prevalence, polymelia, polydactyly, micromelia and syndactyly were registered. Snails of the family Planorbidae are intermediate hosts in the life cycle of *Robeiroia* [49], and the presence of these snails in the diet of *A*. *ordinarium* [14] indicates that these snails are present in at least some of the streams inhabitad by this salamander. This finding suggests the possible implication of parasites in the incidence of some of the abnormalities registered. Additionally, changes in water quality caused by human activities favor a proliferation of planorbid snails [11].

The increasing prevalence of morphological abnormalities has recently been suspected as a potential factor implicated in the decline of amphibian populations and species [1]. To our knowledge, this is the first field study that shows a direct association between the incidence of morphological abnormalities and the quality of the habitat in an endangered salamander throughout its entire distribution range. Therefore, our results suggest that one of the several negative effects of habitat degradation on amphibians is an increase in morphological abnormalities with marked consequences for the survival and general fitness of aquatic amphibians. Studies evaluating body abnormalities can provide important information for determining the causal elements and can be a sign of the health status of the ecosystem, since the proliferation of body abnormalities can not only cause death to individuals but may be a warning sign of environmental degradation [50, 51, 52].

## Supporting information

S1 Fig Pictures showing habitat condition a) optimal, b) suboptimal, c) marginal, and d) poor.
(TIF)

S1 Table Data set underlying findings in this study. (DOCX)
