# Supplementary figures and images for: Habitat quality affects the incidence of morphological abnormalities in the endangered salamander Ambystoma ordinarium
Source: PLoS One. 2017 Aug 28;12(8):e0183573. doi: 10.1371/journal.pone.0183573 (PMC5593498; doi:10.1371/journal.pone.0183573)

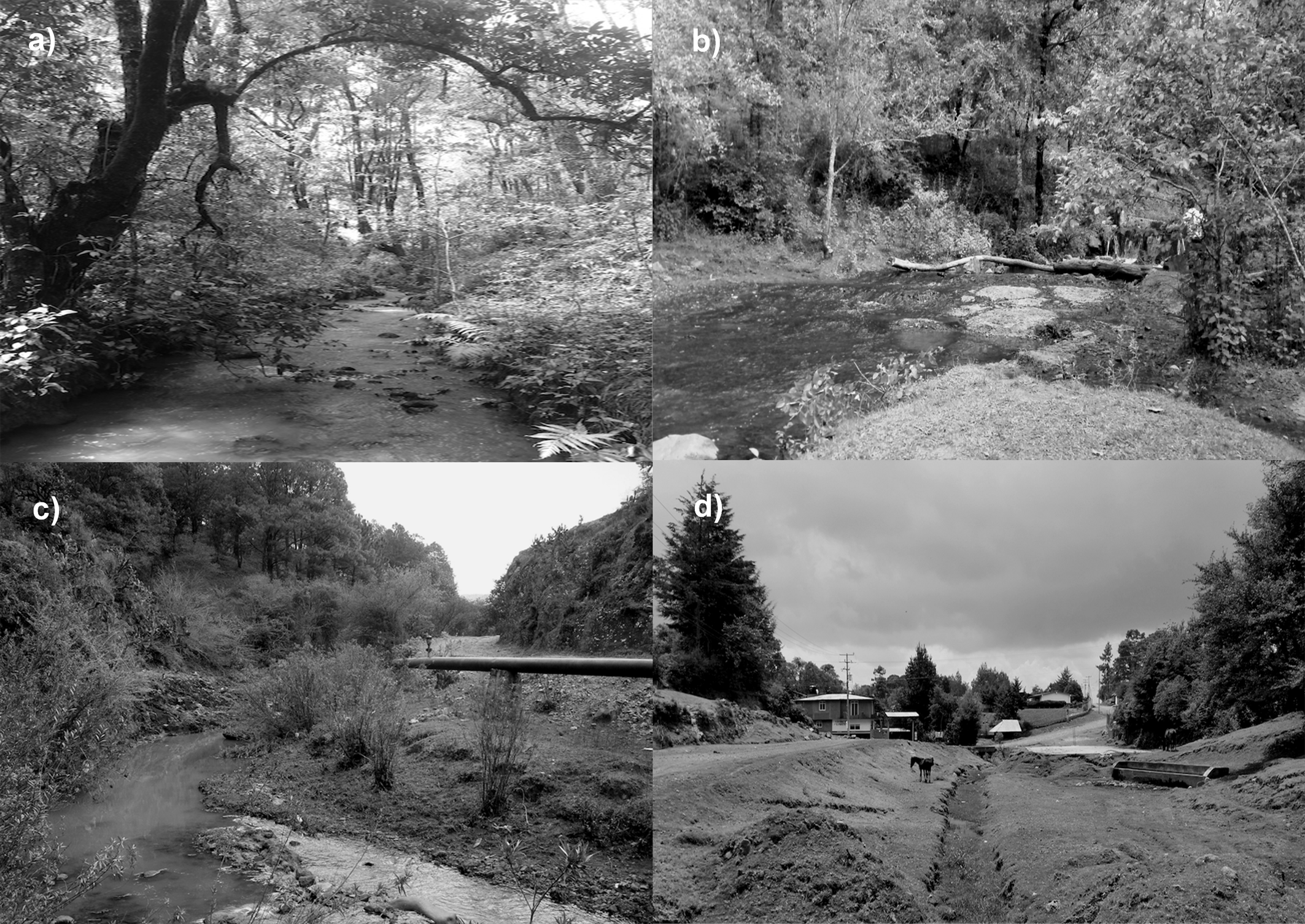

Supplement: S1 Fig — Pictures showing habitat condition a) optimal, b) suboptimal, c) marginal, and d) poor. (TIF) [file pone.0183573.s001.tif]
