# Supplementary material for: Habitat quality affects the incidence of morphological abnormalities in the endangered salamander Ambystoma ordinarium
Source: PLoS One. 2017 Aug 28;12(8):e0183573. doi: 10.1371/journal.pone.0183573 (PMC5593498; doi:10.1371/journal.pone.0183573)
Supplement: S1 Table — (DOCX) [file pone.0183573.s002.docx]

S2. Data set underlying findings in this study

| Location | Number of individuals | Number of abnormal characters | Number of normal characters | Scores of RBPs | Classification of habitat condition |
| --- | --- | --- | --- | --- | --- |
| AGO | 45 | 26 | 495 | 156 | Suboptimal |
| AGZAR | 64 | 29 | 704 | 153 | Suboptimal |
| CMP | 50 | 71 | 550 | 63 | Marginal |
| CP | 22 | 21 | 242 | 154 | Suboptimal |
| CPZ | 21 | 9 | 231 | 154 | Suboptimal |
| CSJC | 78 | 33 | 858 | 172 | Optimal |
| GOL | 22 | 3 | 242 | 123 | Suboptimal |
| KM | 9 | 13 | 99 | 81 | Marginal |
| LNZ | 10 | 11 | 110 | 75 | Marginal |
| PR | 13 | 18 | 143 | 125 | Suboptimal |
| RBC | 32 | 15 | 352 | 178 | Optimal |
| RBP | 80 | 68 | 880 | 105 | Marginal |
| REJ | 13 | 4 | 143 | 110 | Marginal |
| SAVL | 5 | 7 | 55 | 134 | Suboptimal |
| TUR | 26 | 6 | 286 | 157 | Suboptimal |
| VAQ | 12 | 0 | 132 | 145 | Suboptimal |
